# Management of Diabetes Mellitus in Refugee and Migrant Patients in a Primary Healthcare Setting in Greece: A Pilot Intervention

**DOI:** 10.3390/epidemiologia2010002

**Published:** 2021-01-04

**Authors:** Anastasia I Kolomvotsou, Elena Riza

**Affiliations:** 1 Dietetic Department, Polyclinic of Olympic Village, 13672 Αxarnai, Attiki, Greece; 2 Department of Hygiene, Epidemiology & Medical Statistics, School of Medical, National and Kapodistrian University of Athens, 75 Mikras Asias, 11527 Athens, Greece; eriza@med.uoa.gr

**Keywords:** refugees, diabetes, self-management, group sessions, nutrition, primary healthcare

## Abstract

Over the past years there is a substantial wave of migrants and refugees all over the world. Europe accepts approximately one-third of the international migrant population with Greece, in particular, having received large numbers of refugees and migrants by land and sea since the beginning of the civil war in Syria. Diabetes, a non-communicable disease, is a global health problem, affecting people in developing countries, refugees and migrants, and its basic treatment tool includes self-management and education. In this pilot study, we organized educational, interactive group sessions for diabetic refugees, based on culture, health, and nutritional needs according to a questionnaire developed for the study. The sessions were weekly, for two months, in the context of primary healthcare, organized by a dietitian. Nine individuals completed the sessions, five of nine were diagnosed in Greece and seven of nine needed diabetes education. Their waist circumference was above normal and they were all cooking at home. Their nutritional habits improved by attending the sessions and the interaction helped their social integration. They all found the sessions useful, and felt more self-confident regarding diabetes control and healthier.

## 1. Introduction

Since 2015 a huge wave of migrants and refugees moved all over the world in the search of a better future. Europe accepts approximately one-third of the international migrant population, which in present accounts to 72.6 million [1]. The majority of forcibly displaced populations in Europe come from Syria, Afghanistan, and Somalia with Greece being a first entry country into the European Union, given its geopolitical position, hosting over one million migrants crossing its borders.

Non-communicable diseases (NCDs) are chronic diseases which result from a combination of genetic, physiological, environmental, and behavioral factors. Major NCDs are diabetes, cardiovascular disease, chronic respiratory disease, and cancers. They are an increasing health challenge worldwide [2], an important cause of premature death, also prevalent in developing countries, where over 70% of these deaths occur, hereby affecting the health of refugees and migrants [3]. According to data from 2015, NCDs account for 19%–46% mortality in the top five source countries of refugees and migrants [4]. Moreover, the health of refugees and migrants is adversely affected by conditions that prevail in their home country, by certain conditions during their displacement journey and by the adjustment in the host country [5]. Migrants and refugees are in need of health care upon their arrival, but also during their resettlement and integration period. In addition, the effective management and treatment of patients with NCDs requires access to the health care systems and to service providers for emergency needs as well as for medical follow-up [6]. The increased NCDs prevalence together with the massive growth in migration have created the need for effective interventions in primary health care in the host countries [7].

Diabetes mellitus (DM), or Type 2 diabetes, is one of the most common NCDs and its prevalence has become a global health problem [8]. Diabetes is a serious disease for refugees and migrants may be due to genetic profiles, lifestyle, and utilization of the health care system in different ways, and it constitutes a chronic disease of a high prevalence in their home countries, and is also associated with exposure to several risk factors present during their resettlement. Refugees and migrants are considered vulnerable population groups, often lacking access to health services, to essential medicines and do not purchase healthy foods because of increased cost. WHO has set goals to systematically integrate policy and action to reduce health inequalities and tackle NCDs, through health promotion and disease-prevention programs addressing groups of high risk individuals so to maximize population coverage in treatment and care (WHO). Furthermore, preventing and controlling NCDs is significant for achieving the three pillars of economic growth, social equity, and environmental protection with the ultimate goal of achieving sustainable development. The successful management of DM is linked with a clear understanding of the diagnosis of the disease by the patient, its complications and the regulated control of blood glucose levels. Controlled diet, regular exercise, and proper medication are essential parts in the management of the disease together with access to specialized and continuous care health services.

Furthermore, the self-management of DM requires active control from the patient to follow a suitable diet, regular exercise and correct medication in order to prevent complications. As such, patient education on disease self-management and professional support are regarded as critical elements of treatment for all diabetic people and form a major tool in the first line management of the disease which normally takes place in the primary healthcare service setting [9]. The goals of diabetes education are to improve metabolic control, to prevent the development of acute and chronic complications, and to generally increase the quality of life with the disease [10].

The aim of the present study was to organize educational groups for diabetic refugees and migrants taking into account their cultural characteristics, health literacy level, and nutritional needs, in the context of primary healthcare, and to assess the efficacy of the intervention in various factors from the participants themselves. This approach could serve as an additional approach to improve diabetes control and to provide evidence for implementing such group sessions in primary healthcare settings. The increase of self-confidence in the management of their disease through educational material during the sessions, group interactions as well as interactive time with healthcare professionals were evaluated at the end of the sessions. Generally, education, changes in dietary and lifestyle behavior, improving self-management of diabetes, wellbeing and participation were evaluated, in the context of primary health care provision for refugees and migrants.

## 2. Materials and Methods

Our study was a health promotion intervention for diabetic refugee and migrant patients in a primary healthcare setting in which we focused on assessing evidence in three areas: (a) the degree of attendance, interaction and active participation in a group of diabetic patients, (b) the level of knowledge of the patients on their disease, its management and its complications, and (c) the degree of satisfaction on the self-management of DM after the intervention.

In order to assess the level of knowledge of the diabetic refugee and migrant patients, we collected information with the use of an interviewer administered questionnaire (Appendix A) specifically designed for this study. The interview was conducted by a dietician with the help of a translator in the patient’s language and it also included anthropometric measurements. Data were collected to assess the knowledge of the participants on DM, the changes in their nutritional habits before and after their displacement, their living conditions, their physical activity levels, and their general health status. According to the confidentiality operating policy of the primary healthcare clinic and in order to increase the trust of vulnerable individuals (such as refugees, migrants, homeless) in using the service, no socio-demographic characteristics, educational level, or occupation were recorded. In the anthropometric measurements we measured height, weight and waist circumference in duplicate and the recorded values were averaged. Height was measured to the nearest centimeter barefoot using a portable stadiometer. Weight was measured in kilograms to the nearest decimal without shoes and in light clothes using a flat weighing scale. Waist circumference was measured to the nearest centimeter at the middle between the iliac crest and the rib cage using a measuring tape. Body mass index was calculated from weight and height as kg/m^2^. Furthermore, participants were asked to keep a 24 h dietary recall in order to provide information to allow discussion on their daily food with the dietician.

In order to assess the confidence in and motivation of the participants for managing DM after the intervention, two cultural mediators were asked to participate in a joint meeting at the end of the intervention to facilitate the discussion with the refugees and migrants in their native languages, Arabic and Farsi.

### 2.1. Recruitment, Enrollment, and Baseline Characteristics of Participants

A total of 11 individuals with DM who had visited the outpatient clinic of the primary health care setting were initially recruited (five from Syria and six from Afghanistan). Two (2) individuals dropped out after the first session. These patients were often attending this NGO managed outpatient clinic for refugees, migrants and other vulnerable population in the city of Athens. They were all taking medication for diabetes. After being informed, they consented to attend the sessions and agreed to fill in the questionnaire regarding their knowledge on their disease, their concerns, their need for medical assistance, their nutritional habits, before and after their displacement, their living conditions and their health status. The youngest participant was 23 and the oldest was 60 years old, three women and eight men. Written informed consent was obtained from each participant prior to the beginning of the sessions.

### 2.2. Intervention

**Figure 1:**
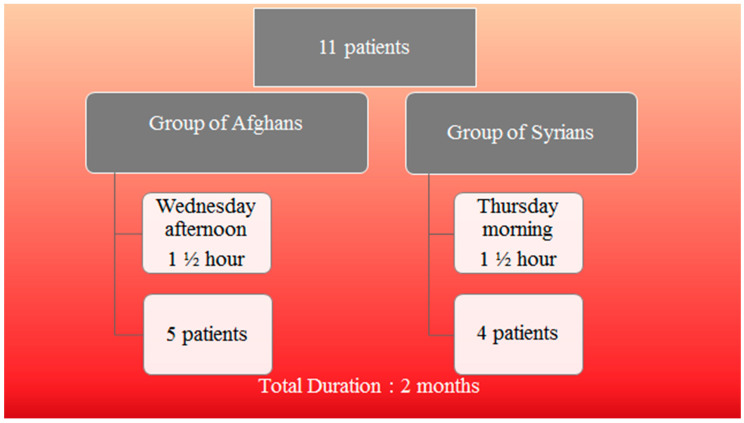
Organization of the intervention.

Two groups of patients were formed based on country of origin, four from Syria (one woman and three men) and five from Afghanistan (two women and three men), to allow for better communication in terms of language and culture within each group. The group of Afghans was attending the diabetic group seminars every Wednesday afternoon and the group of Syrians every Thursday morning (Figure 1). The weekly seminars of the diabetes education lasted 90 min for a total duration of two months. The first part of the sessions was dedicated to presenting the clinical aspect of the disease and was done by a nurse specialized in DM. The nurse gave the participants useful information on the disease, the symptoms, the complications and the use of medication. This part was complementary to our intervention session, it will not be analyzed further, but it was essential for the patients. The second part of the sessions was delivered by a dietitian, specialized in diabetes. The dietician presented information on elements of nutritional therapy in relation to DM. The sessions were conducted in the language of each ethnic group (Arabic for Syrians and Farsi for Afghans) to allow for cultural adaptation where necessary. Each week the dietician analyzed a different theme. During the sessions, the patients of each group had the chance and were prompted to interact with each other. There was time to share personal experiences regarding DM. Questions were also posed by the two specialized group educators (the nurse and the dietician) relating to the subject of each session. At the end of the session, patients took part in interactive activities related to the week’s theme of education, in order to practice, to experiment and to apply the new information. The type and content of the educational material used was adapted to their demographic characteristics, their living conditions including economic status, their specific nutritional habits and their way of living influenced by their culture and traditions.

Regarding the education on how to make an appropriate daily dietetic plan, participants were asked to bring a 24-h dietary recall to discuss with the dietician and to assist in the formation of their daily eating pattern. All the sessions were organized taking into account the patient’s wellbeing, in terms of physical and mental health and the material was delivered (written and spoken forms) in a tailor-made, comprehensive manner, taking into account the levels of literacy and health literacy in each group.

The aim of the dietician’s contribution in the diabetic group sessions was to educate the patients on issues related to their disease aiming at controlling their blood glucose levels, at staying healthy and at preventing diabetic complications. Moreover, action was taken to help them familiarize themselves with the food products of the host country, Greece, in an effort to make the adaptation in the new environment smoother. Furthermore, taking part in group sessions was important for their socialization, kept them busy in a weekly activity, and gave them a sense of belonging.

The dietetic part included information on the definition of energy that derives from the consumption of food, on macro and micronutrients, on food groups, on the glycemic index, on the Mediterranean diet, on the formation of a diabetic plate, on portions, meals, and on making daily and weekly dietetic plans, on salt and sugar contents and on food labels. Further information was given on the importance of maintaining a healthy body weight, the association of waist circumference and physical activity in the control of diabetes, on diet during special periods like Ramadan and on providing tips for the diabetic patient when travelling. All the sessions were adapted to the culture, the economic status and the living conditions of the participants, based on the information provided in the questionnaire they had filled in at the beginning of the intervention concerning their nutritional habits at the starting point of the intervention, before and after their displacement, their living conditions, and their general health status.

### 2.3. Data Analysis

Descriptive statistical analysis was only performed. Multivariate analysis was not performed as it presented substantial methodological obstacles because of the small sample that resulted in non-accurate model estimate parameters. Moreover, the sample used did not provide a good representation of the refugee population. However, as this health promotion intervention was a pilot study to assess the effectiveness of the intervention to groups of refugees and migrants in terms of content and culturally adapted information for DM management, the combined quantitative-qualitative interpretation of the data can be used.

## 3. Results

Out of the 11 individuals initially recruited, two patients suddenly stopped attending the group sessions and they did not visit the outpatient department again. A total of nine patients completed the sessions, three women and six men, showing high acceptance and satisfaction of the group sessions, mentioning that they were feeling relaxed and comfortable (Table 1). According to the questionnaire; four were diagnosed with DM prior their arrival in Greece while five were diagnosed with diabetes in Greece and had already some knowledge on their disease. Two out of nine had some knowledge on the general recommendations to follow for DM, despite the fact that six of them already had the experience of a family member also diagnosed with diabetes. Most of them were seeing a doctor once a month (five patients) for different reasons and their concern about DM was mostly the disease itself, their body weight (two patients), fertility and sex problems (two patients) and vision problems (one patient).

Only one of the patients was still living in a reception and identification camp (RIC), the rest had been settled in permanent accommodation (such as apartments, houses). All of them were preparing their food daily themselves. They mentioned some changes in their eating habits since living in Greece (Table 2). More specifically, they were consuming fish and meat less frequently than before mostly due to economic restraints and they had also decreased the number of daily meals. They continued drinking tea and water, although they decreased the quantity of water due to the taste of water and to their inability to buy bottled water. After the sessions, they mentioned having started consuming less milk and yoghurt, compared to what they used to according to their culture and they reduced the consumption of nuts. They started using olive oil and had increased the consumption of vegetables. Generally, most of them mentioned that they were eating healthier after the sessions on diabetes.

Regarding their physical activity levels, six participants reported walking one to two hours daily, two were walking 4–5 h daily and one half an hour to one hour.

The anthropometric measurements showed that five patients were obese with a mean BMI of 35 kg/m^2^ (range of 33–36.5 kg/m^2^), one was overweight with a BMI of 27.4 kg/m^2^ and three were in the normal weight range (mean BMI 23.7; 22.5–24.8 kg/m^2^). These results indicate that this is a high risk group for type 2 diabetes in need of careful management. Their waist circumference was above normal range both for women (>88 cm) and for men (>102 cm). Based on the questionnaire responses at the beginning of the sessions, seven patients aimed to reduce their body weight and five succeeded to achieve a weight loss by the end of the two-month intervention period.

During the final session, the patients were asked to provide feedback and to make comments on the total experience from the sessions. They all reported finding them really useful, interesting and of sufficient duration. The interactive part at the end of the group session in particular, contributed to a high degree of interest that was maintained during the whole duration without difficulty to follow the material at any point. When asked about potential changes in the organization of the sessions, they generally suggested to increase the duration of each session, to include additional attendees in the group to boost their socialization, to include information for psychological support and to provide additional practical information on healthy cooking. They evaluated positively the “homework” set for them after each session. They all mentioned feeling more self-confident with the control of DM than before the sessions and that they were feeling better and healthier (Table 3).

## 4. Discussion

NCDs are an increasing health problem present both in developed and in developing countries. Refugees and migrants are exposed to risk factors during the migration journey or resettlement which may be linked to NCDs such as DM. Thus, treating NCDs in these population groups has become a goal for humanitarian health.

According to data from the surveillance system in Greece, the most common causes for medical consultation in refugees and migrants were infections of the respiratory tract (23% of the total) and DM in 27% [1]. DM is a common and serious disease in refugees and migrants since it has a high prevalence already in their home countries, where due to wars or poverty it might remain undiagnosed or inadequately treated. This population at the countries of resettlement, like in Greece, should receive medical treatment in emergency settings, as a short-term solution, for complications that might relate to DM, but most importantly they should be offered the opportunity to access specialized health care for long term medical care and public health support [11].

Primary health care for diabetes requires different specialties for the provision of health care and specific orientation of the health systems [12]. Health education focusing on diet, exercise and medication are an effective tool for the management of DM in primary health care settings.

To our knowledge, this is the first study of delivering group sessions to diabetic refugees and migrants in a primary healthcare setting. It is a pilot study and the first to show that providing tailor-made medical and dietetic education for the management of diabetes in groups of vulnerable populations is feasible and highly effective. The intervention was perceived as very interesting and useful from all participants.

The two-month duration of the intervention is in accordance to the duration of diabetes self-management interventions also mentioned in other studies. Hung et al. (2017) showed that seven educational sessions for a duration of two months were effective in long term as indicated from measurements of blood glucose levels and from assessing the diabetic health literacy of diabetic persons and could be a reference for other similar interventions for DM patients [13].

The use of questionnaires at the beginning of the study in the context of an interview with the dietitian and the help of an interpreter provided information on very important study factors regarding knowledge on the disease, concerns about diabetes and need for medical assistance. Furthermore, important information on the nutritional changes due to displacement, living conditions and health status helped the session organizers to satisfy the patients’ needs in terms of cultural adaptation and practicality and facilitated use of the educational tools in their everyday life. As shown in a another study, the use of a diabetes-specific questionnaire, individualized for measures of quality of life of such patients, was a tool that could be used even in clinical settings for clinical research and to create educational programs as it provides a valuable, in-depth understanding of the patients’ needs and can be effective in prioritizing and specifying the educational material [14].

Most of the patients were diagnosed in Greece and were unaware on the details of their disease, although some of them had family members with diabetes, as also shown in another study where the diagnosis of the disease of the refugees and migrants was made after their arrival in USA [15].

Two different specialized professionals on DM designed and delivered the diabetic group sessions: a nurse formed the medical part on self-management of DM and a dietitian the nutritional and the dietetic part. This is in accordance with the instructions of the National Standards for Diabetes Self-Management Education and Support [16] that require at least one member of staff in the provision of diabetic self-management education to be specialized instructor(s) on diabetes and of the profession of a registered nurse or a registered dietitian. As these two professions are the main providers of diabetic self-management education, their allocation as educators is also strongly supported and can be used for group sessions in clinical settings even with limited resources [9].

The structure of the dietetic sessions included key messages for the diabetes self-management. Other information given during the sessions referred to nutrients, food groups, glycemic index, dietary pattern, portions and meals. Body weight and physical activity were recorded. The patients were getting a stronger idea of the important link between diabetes management, correct portions and physical activity in combination with the correct food choices, in accordance with the aim of the WHO to increase levels of awareness regarding the disease and diabetes complications especially in patients from developing countries [7]. Furthermore, in a study on innovative ways for the self-management of DM [17] the key main messages for the changes in their behavior, were dietary quality and physical activity. In this study, as in our pilot study, the diabetic patients had a positive reaction to change based on this information. Furthermore, as in our study, the educational material included in the sessions was culturally based, giving advice for the diet during Ramadan and tips for travelling with diabetes and was provided with the help of a translator. DM educational material taking into account the peculiarities of a specific ethnic group to increase effectiveness in the management of diabetes, was also highlighted in a review regarding self-management of diabetic adults in the Gulf Cooperation Council countries [18]. Furthermore, culturally based educational material was shown to increase health monitoring for refugees with multiple chronic conditions in another study [19]. The information on dietetic sessions was also based on socioeconomic status, lifestyle and cultural, and traditional food choices in another study [18].

Most patients in our study mentioned losing weight during the two-month period of the sessions denoting effective self-management education [13]. Physical activity was included in the diabetic educational material; however, as most of the patients in our pilot study used to walk a great deal, this was not a factor affected by the sessions. The walking habits of the patients in our diabetic group sessions can be justified by their current lifestyle, which is characterized by restricted use of public transport due to economic reasons and by unemployment which is linked with large amounts of free time during the day.

An important finding according to the diabetic patients in our study during the sessions is that there was no change regarding the choice and consumption of fast foods. This is in contrast with other studies that show increased frequency of refugees and migrants in adopting a new, unhealthy lifestyle, characterized by fatty foods, fast food choices, physical inactivity, resembling the majority of the general population of the host country with a corresponding increase in the risk for cardiovascular disease and development of DM [10]. It seems that eating habits based on their traditional eating, which includes consumption of legumes and rice and dairy products and chicken, as well as the sessions about healthy eating favorably influenced their food choices. Specifically, basic changes mentioned by the patients was increase in vegetable, and olive oil consumption and decrease in the consumption of nuts and full fat dairy products for every meal, all of which define a healthy dietary plan, as it was explained in the sessions. However, it was mentioned that the reduced consumption of fish and meat was due to financial problems and not by choice.

The high level of participation in the diabetic group sessions is also an index of the need for easily accessible, comprehensive and culturally-based programs in the primary health care sector. Refugees and migrants with DM often have limited access to health care services, thus they fail to follow the relative recommendations for disease management and they from diabetic complications [12]. Their living and societal conditions [20] the high levels of illiteracy [21] and cultural barriers regarding the management of chronic diseases [22] are often factors that influence the progression of the disease, through improper diet, reduced physical activity levels and poor medication compliance [23]. Partnerships between refugee, migrant service organizations, and primary health care service providers are needed for the standardization of best practices in DM management [24] Succeeding in helping the refugees and migrants with chronic diseases to remain healthy will also positively affect their new living environment, the re-establishment of themselves and of their families and will meet the needs of the community [3].

In the final session, the patients reported finding the diabetes group sessions useful and interesting. All mentioned feeling more self-confident with the control of diabetes than before the sessions, due to the increased information on their disease and that they were feeling better and healthier. Patients who are able and confident to better manage their disease fulfill the basic component in diabetes care. Intervention in diabetic refugees and migrants on the basis of educational messages in primary health care in the study of Mark et al. showed an improvement in healthy self-management behaviors [17].

Regarding the evaluation at the end of the group sessions, the needs of the refugees and migrants for lessons on healthy cooking are consistent with the suggestion of the study of Yun et al., for education on healthy food preparation and on healthy eating habits. In that study, these targets were set for primary care and community health education even in newly arrived refugees and migrants [3].

The positive effects observed in our study are also reported in a systematic review [25] indicating that education and culturally focused interventions in primary health care, improve diabetes management, decrease disease complications and can be included in clinical settings overcoming problems like limited resources [9] or restricted access to such programs [26].

Given the high prevalence of NCDs in refugees and migrants, further research on the health concerns and needs of this population during the early resettlement period is required, along with the need for additional diagnostic tools and screening protocols for the identification of refugee status [27]. In the US, refugee-related research focuses in communicable diseases [28], whereas in Greece there is need for research on the role of health care use in diabetes management and the clinical management of DM as this information can help to organize effective interventions. Implementation and evaluation of new, evidence-based models of care are required in order to provide culturally sensitive disease self-management support in primary care [9].

This pilot study is a model example of primary health care for DM in refugees and migrants indicating that chronic diseases, such as diabetes can be addressed through culturally based education, self-care support, changes in lifestyle behavior and control of possible diabetic complications. This health care intervention when provided in groups, can contribute to their societal integration and their adjustment in the new country of residence. This pilot study can be a model practice of primary health care delivery nested in humanitarian health programs for refugees and migrants that can be incorporated into national health systems.

### 4.1. Strengths

This pilot study is a model heath education intervention for diabetic refugees. The positive characteristics are the culturally tailored element, with swift and careful recruiting process, high quality, updated educational material, and well-planned and effectively conducted group education with interactive time.

### 4.2. Limitations

The study participants were not randomly selected, since there was no formal registration of the outpatient clinic for diabetics and there is no follow up or obligation to return. As such, there was no defined sampling frame to randomly select any study participants and the number of diabetics in the clinic that could be identified was very small. This is possibly due to difficult access to this clinic as they reside far away from the city center, or because they visit the NHS facilities instead, or they do not perceive the need to visit any medical facility altogether or possibly due to inability to recognize the symptoms. The total number of participants was small and there was no possibility to perform any biochemical tests due to insufficient resources.

## 5. Conclusions

Although most of the migrants and refugees in Europe are in a good health, some face medical problems in addition to economic and social difficulties. European healthcare delivery services should accommodate the management of any disease, especially chronic diseases. Apart from the provision of treatment in refugee and migrant camp settings there is need for effective programs at the primary healthcare level as they will positively affect the health of refugees and migrants, their adaptation to the new country and will also promote the public health of the general population in host countries.

Although further studies are needed with larger number of participants, additional evidence-based models of care for DM management for refugees and migrants in primary health care, the results of the current pilot study are encouraging. This health promotion intervention in a primary healthcare setting showed that participation of diabetic refugees in-group sessions is feasible and enhances the ability of these people to learn and to apply the delivered information provided with respect to their culture, taking into account their living conditions and socioeconomic status. Diabetic group sessions addressing refugee and migrant patients in primary health care can become standard practice as they resulted in healthier changes in some nutritional habits and in an increase in self-management of diabetes that could help to reduce diabetic complications, to improve their quality of life and their adaptation process in their new environment.

## Figures and Tables

**Table 1:** Characteristics of the population.

Country of origin	Syria and Afghanistan
Total number participants	9
Gender	3 women (Syria 1, Afghanistan 2) 6 men (Syria 2, Afghanistan 2)
Age range	23–60 years
Mean body weight	91.5 kg
Mean BMI	30.4 kg/m^2^
BMI classification	5 obese, 1 overweight, 3 healthy weight
DM diagnosis	5 diagnosed in Greece
DM in the family	6 had a family member with DM
Housing	8 accommodation in flats 1 accommodation in reception camp
Cooking habits	Home cooking

**Table 2:** Changes in eating habits after nutrition education sessions.

Changes in Nutritional Habits	Etiology
Less fish & less meat	Economic reasons
Less number of meals	Economic reasons
Less water	Economic reasons for bottled water, they did not like the taste of tap water
Reduced consumption of milk & yoghurt (cultural habits)	Nutrition education for DM
Reduced consumption of nuts	Nutrition education for DM
Increased consumption of olive oil	Nutrition education for DM
Increased consumption of vegetables	Nutrition education for DM
Healthier eating habits overall	Nutrition education for DM

**Table 3:** Seminar evaluation and proposals for future meetings.

Seminar Evaluation	Proposals
Useful and interesting	Increase the duration of each session
Enjoyable, especially the interactive part at the end of the each group session	Increase the number of attendees in each group, so to help with their socialization
They felt more self-confident with the control of diabetes, than before	Include psychological sessions
They were feeling better and healthier	Include practical sessions on healthy eating

## Data Availability

Data from this study are safelly secured by the primary researcher, are anonymous and maybe availlable upon request.

## Appendix A

**Table A1:** Questionnaire.

**1**	**Do you know about diabetes?**	**Yes No**
**2**	**The 1st time you were diagnosed with DM was in Greece?**	**Yes No**
**3**	**Does anybody else in your family has diabetes?**	**Yes No**
**4**	**Are you familiar with the recommendations for the therapy of diabetes?**	**Yes No**
**5**	**Has your body weight changed from when you came to Greece**	**Same Increased Reduced**
**6**	**Have you tried to change your body weight and if yes for what reason?**	**To increase To reduce**
**7**	**How many times per week do you eat in a camp**	Everyday
		2–3 times/week
		Less than once/week
	**In a restaurant**	Everyday
		2–3 times/week
		Less than once/week
	**At home**	Everyday
		2–3 times/week
		Less than once/week
**8**	**How often do you cook and where?**	
**9**	**What kind of food did you use to cook more frequently when you were in your country?**	**What kind of food do you use to cook more frequently in Greece?**
**10**	**How many meals did you have before?**	**How many meals you have now;**
	breakfast	breakfast
	snack	snack
	lunch	lunch
	afternoon snack	afternoon snack
	dinner	dinner
**11**	**What did you use to eat every day in your country?**	**What do you use to eat every day in Greece?**
	Dairy products (milk, yoghurt, cheese)	Dairy products (milk, yoghurt, cheese)
	Vegetables raw/cooked	Vegetables raw/cooked
	Fruits/fresh juices/canned	Fruits/fresh juices/canned
	Rice/bread/spaghetti/potato	Rice/bread/spaghetti/potato
	Nuts	Nuts
**12**	**Frequency of consumption per week in your country**	**Frequency of consumption per week in Greece**
	**meat**	**meat**
	1–2/week	1–2/week
	1/month	1/month
	**chicken**	**chicken**
	1–2/week	1–2/week
	1/month	1/month
	**fish**	**fish**
	1–2/week	1–2/week
	1/month	1/month
	**legumes**	**legumes**
	1–2/week	1–2/week
	1/month	1/month
	**eggs**	**eggs**
	everyday	everyday
	2–3/week	2–3/week
	1/month	1/month
**13**	**What kind of oil did you use for cooking before?**	**What kind of oil do you use for cooking now?**
**14**	**What kind of drinks did you consume before?**	**What kind of drinks do you usually consume in Greece?**
	Soft drinks	Soft drinks
	tea	tea
	water	water
	juice	juice
	coffee	coffee
	alcohol	alcohol
**15**	**How many small bottles of water did you drink per day in your country? (1 small bottle = 500 mL)**	**How many small bottles of water do you drink per day in Greece? (1 small bottle = 500 mL)**
**16**	**Which of your dietary habits have remained the same from what you used to do back home?**	Number of meals
		Time of meals
		Kind of food
		Portions
		Drinks
**17**	**Did you find the quantity of food you needed before?**	**Do you find the quantity of food you need now?**
	Yes no partly	Yes no partly
**18**	**Did you find the variety of food you needed before?**	**Do you find the variety of food you need now?**
	Yes no partly	Yes no partly
**19**	**The last 30 days did you lose weight because you could not buy food?**	Yes No Don’t know
**20**	**What type of food do you easily find to consume in Greece?**	
**21**	**What kind of foods do you buy in Greece?**	
**22**	**Do you eat fast food?**	**Yes, how many times/week**
		**No**
**23**	**Do you think that the current dietary habits are healthier than the dietary habits you had back home**	**Yes No**
**24**	**How many time do you spent walking every day?**	30 min–1 h
		1–2 h
		More than 2 h
**25**	**Do you have any concerns on your health status?**	Yes, state what No
**26**	**How many times did you visit a doctor during the previous year?**	
